# Aesthetic and Functional Rehabilitation of Phthisis Bulbi Through Custom Scleral Shell Prosthesis: A Technical Case Report

**DOI:** 10.7759/cureus.92676

**Published:** 2025-09-18

**Authors:** Rajesh Shetty, Kevin Lasrado, Sania Hussain, Mallika Shetty

**Affiliations:** 1 Department of Prosthodontics, Yenepoya Dental College, Yenepoya University, Mangaluru, IND

**Keywords:** ocular prosthesis, ophthalmology, phthisis bulbi, prosthodontics, scleral shell prosthesis

## Abstract

This case report presents the fabrication of a custom scleral shell prosthesis for a young female patient with phthisis bulbi resulting from a childhood injury. Phthisis bulbi, an end-stage ocular condition, poses significant challenges due to its effects on orbital anatomy and function. The report describes the procedural steps involved, including impression making, cast fabrication, wax trial, iris orientation and matching, flasking, curing, characterization, and final finishing. Custom scleral shell prostheses are highlighted as a superior option compared to other prosthetic alternatives, as they allow precise customization, provide uniform pressure distribution, and offer improved aesthetics. The discussion emphasizes the importance of prosthetic design, anatomical considerations in phthisical eyes, biomechanical principles, material selection, and the need for customization. This approach not only restores appearance but also enhances psychological well-being and quality of life. The case underscores the need for ongoing interdisciplinary research and innovation to advance ocular prosthetics and improve patient outcomes.

## Introduction

The human eye, often described as the window to the soul, plays a crucial role in sensory perception, emotional expression, and social interaction. Loss of an eye, whether congenital or caused by malignancy, infection, or trauma, such as ocular injury leading to a shrunken, non-functional phthisical eye (phthisis bulbi), creates profound medical, psychological, and social challenges [1]. Patients may experience impaired binocular vision, disorientation, reduced depth perception, and limitations in daily activities, along with emotional effects such as depression, anxiety, and lowered self-esteem. Ocular prostheses have a long history, evolving from ancient Egyptian shell inlays to modern acrylic shells, reflecting advances in materials and anatomical accuracy. Today, custom-made scleral shell prostheses, designed to match individual orbital anatomy, provide superior aesthetics, uniform pressure distribution, and improved psychological adaptation [2]. Despite these developments, progress in ocular prosthetics has not kept pace with other prosthetic fields. This highlights the need for interdisciplinary collaboration that integrates prosthodontic expertise with ophthalmology and psychology. This case report presents the fabrication of a custom scleral shell prosthesis for a patient with phthisis bulbi, demonstrating how modern techniques address both functional and cosmetic deficits, while underscoring the essential role of prosthodontic care in restoring dignity, social acceptance, and quality of life.

## Case presentation

**Figure 1:**
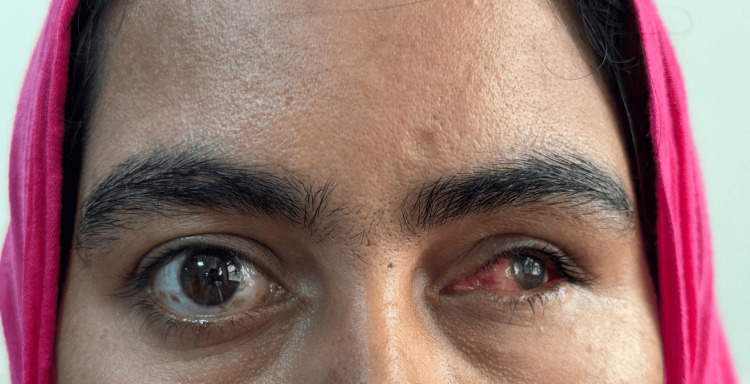
Preoperative image showing the affected eye Note: Written informed consent to include this image in an open-access article was obtained from the patient.

A female patient in her early 20s reported to the Outpatient Department of Prosthodontics and Crown and Bridge with the chief complaint of aesthetic disfigurement due to a scleral defect. She had a history of a childhood fall that led to enophthalmos (a sunken appearance of the eyeball) and subsequent loss of vision in the affected eye (Figure 1). Following a comprehensive clinical evaluation and discussion of treatment options, a custom-fabricated scleral shell orbital prosthesis was planned to restore aesthetics and provide psychological support for the patient.

Procedure

Impression Making

**Figure 2:**
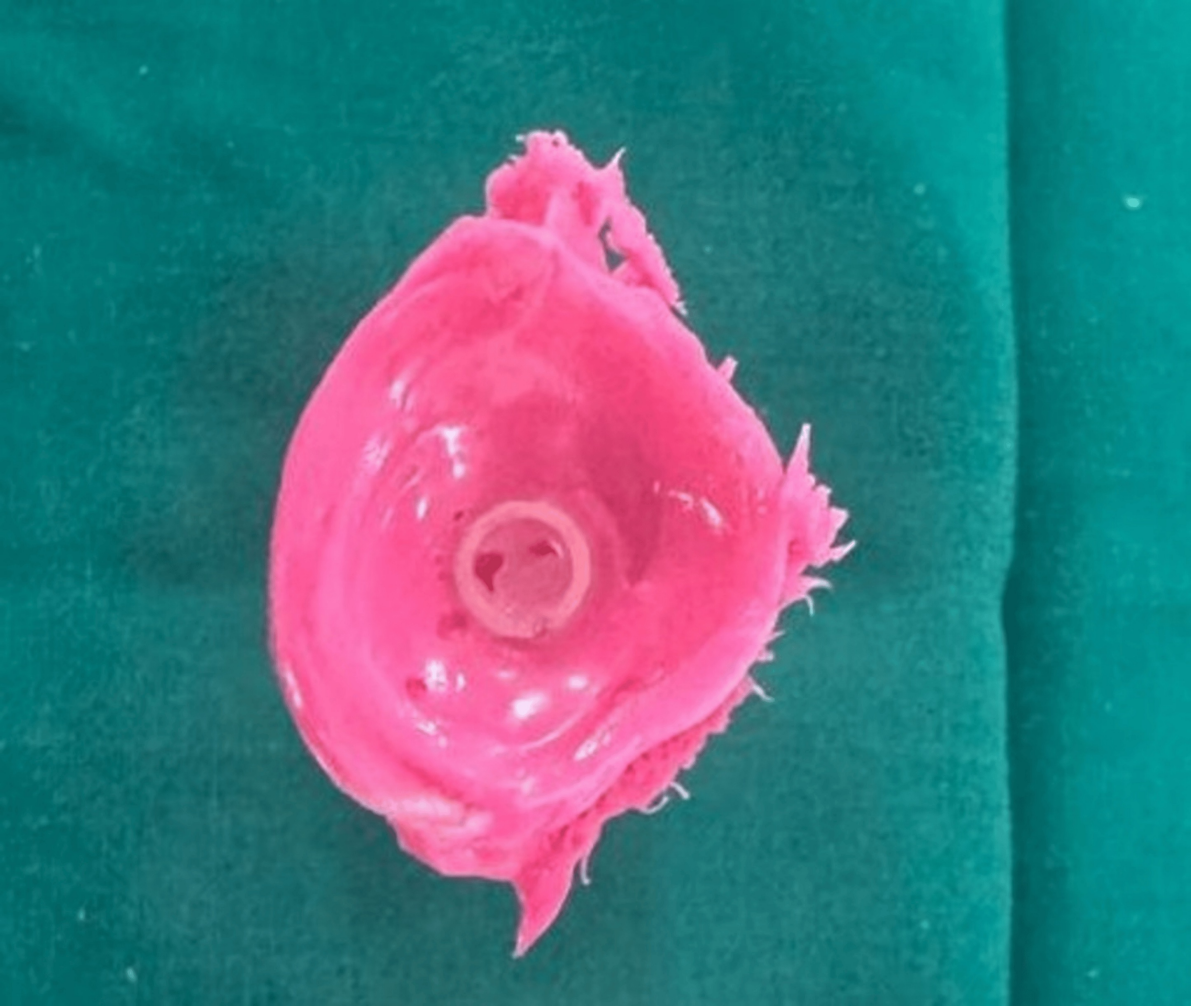
Impression of the defect using light body addition silicone material

An acrylic shell, approximating the size of the patient’s eyeball, was fabricated using autopolymerizing acrylic resin (DPI™). It was then tried in the patient’s eye and adjusted as necessary. The dispenser tip of light body addition silicone impression material (Zhermack™) was attached to the fabricated shell. The patient’s eyebrows, eyelashes, and surrounding areas were lubricated with petroleum jelly to facilitate easy removal of the impression. To record the socket in a functional state, a small amount of impression material was injected into the socket through the shell, and the patient was instructed to move the eye in multiple directions (left, right, up, and down). After retrieval, the impression was carefully examined for defects or voids (Figure 2).

**Figure 3:**
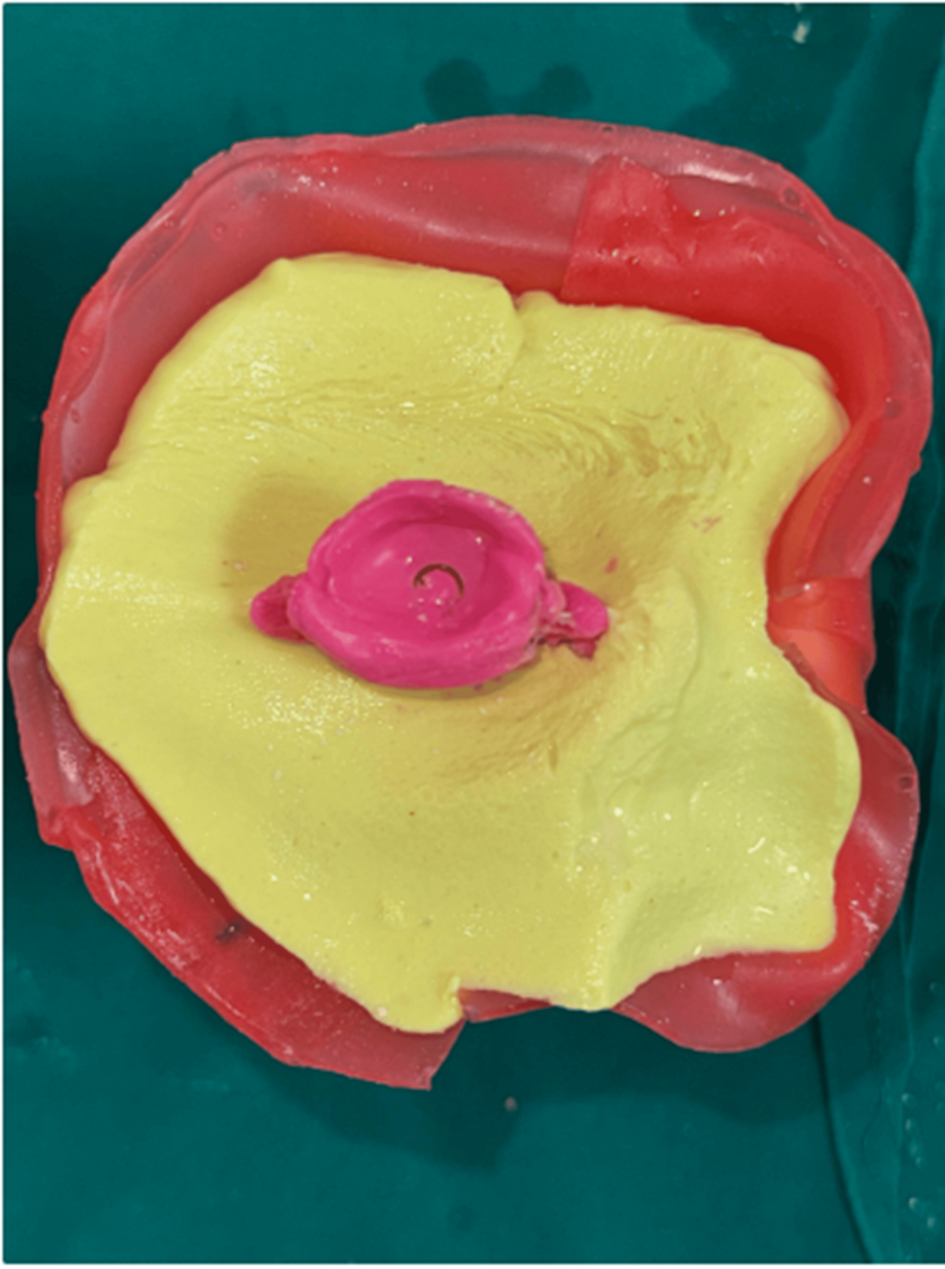
Impression of the surrounding area made and boxed using a modelling wax

Alginate was used to create an impression of the surrounding environment after the impression was reinserted into the eye (Figure 3).

Cast Fabrication

**Figure 4:**
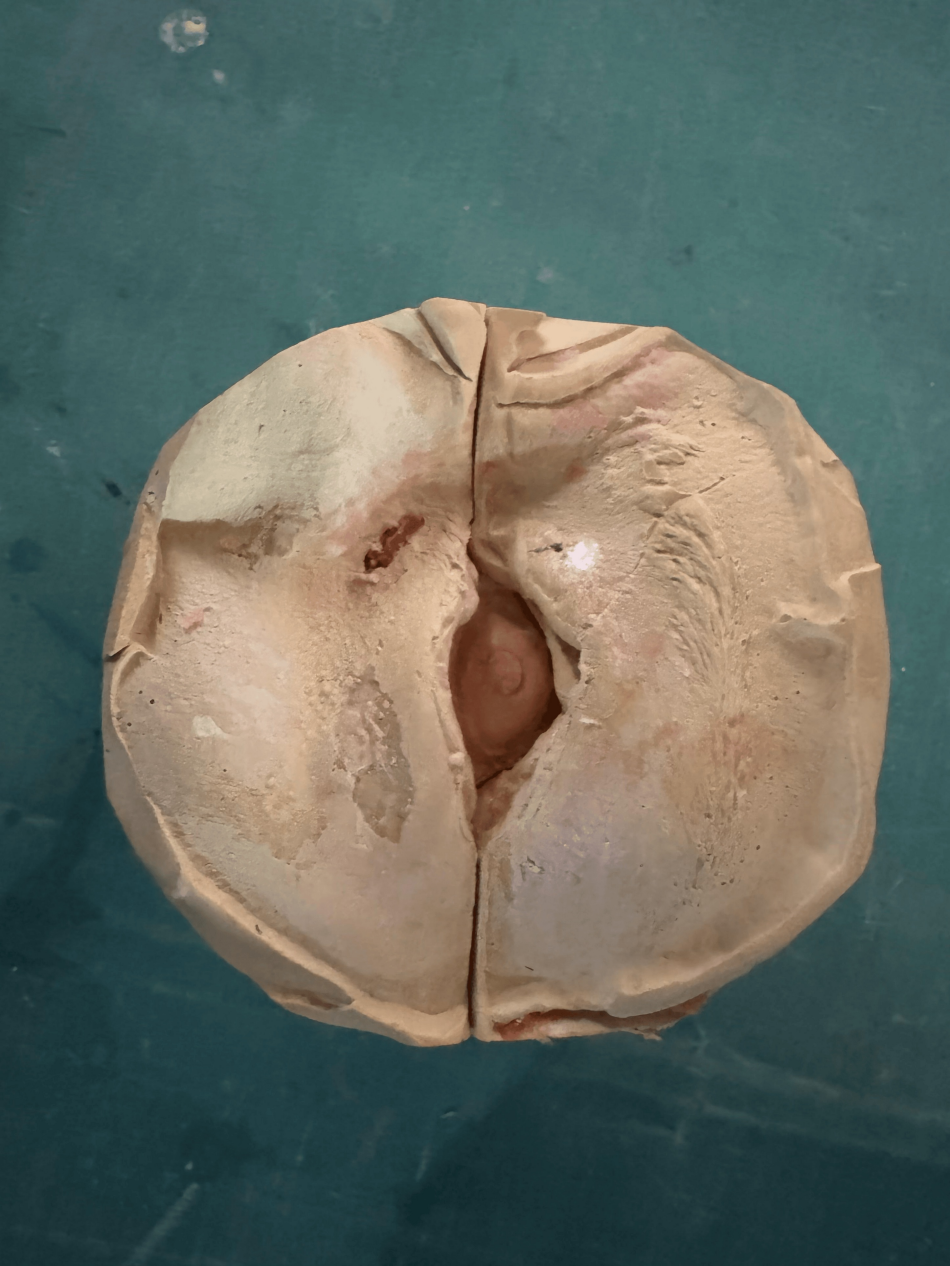
Cast fabrication done using a three-pour technique

The impression was boxed with modelling wax, and the cast was fabricated using a three-pour technique. First, a base layer of dental stone was poured to form the initial foundation. This was followed by a second layer of high-accuracy stone to capture fine details. Finally, a third layer of plaster was added to provide stability and support to the cast. This technique ensures a precise and detailed representation of the patient’s socket anatomy (Figure 4).

Wax Trial

**Figure 5:**
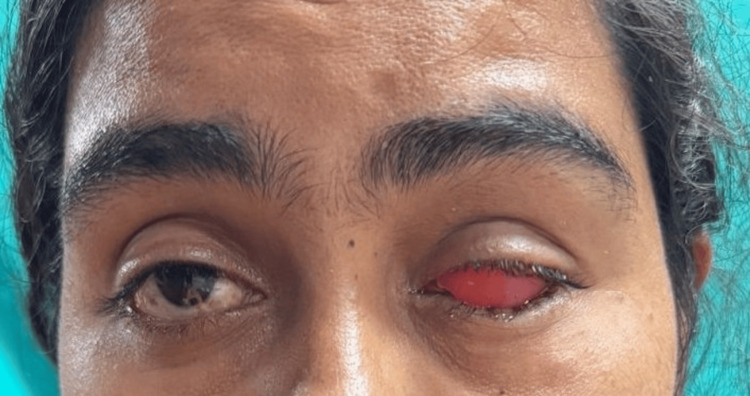
Wax trial done and iris located using a contralateral eye Note: Written informed consent to include this image in an open-access article was obtained from the patient.

A mold was made using putty-type polyvinyl siloxane impression material to replicate the impression. Once the putty had set and the impression was removed, melted white carving wax was poured into the mold. After hardening, the wax pattern was retrieved and evaluated for comfort, extensions, and adequate bulk to restore fullness. The wax pattern was then smoothed and contoured to match the patient’s contralateral eye, ensuring a natural appearance (Figure 5).

Iris Orientation and Matching

The wax pattern was tried in the patient’s socket to evaluate comfort and fit. Three reference lines were marked on the patient’s face to guide iris orientation: the facial midline, a line parallel to the pupil of the unaffected eye, and a corresponding parallel line on the side of the affected eye. The patient was instructed to look straight ahead, and any deviation in iris positioning was corrected. Shade matching was then performed, and a close-up photograph of the contralateral eye was taken to ensure accurate replication of the natural iris.

Flasking and Wax Pattern Curing

Dental stone was used to invest the wax pattern with the iris button in a flask. Dewaxing was then carried out, and colored heat-cure polymethyl methacrylate (PMMA) was packed into the mold. The prosthesis was processed using a long curing cycle (nine hours at 165°F) to ensure complete polymerization and long-term stability.

Characterization and Iris Placement

**Figure 6:**
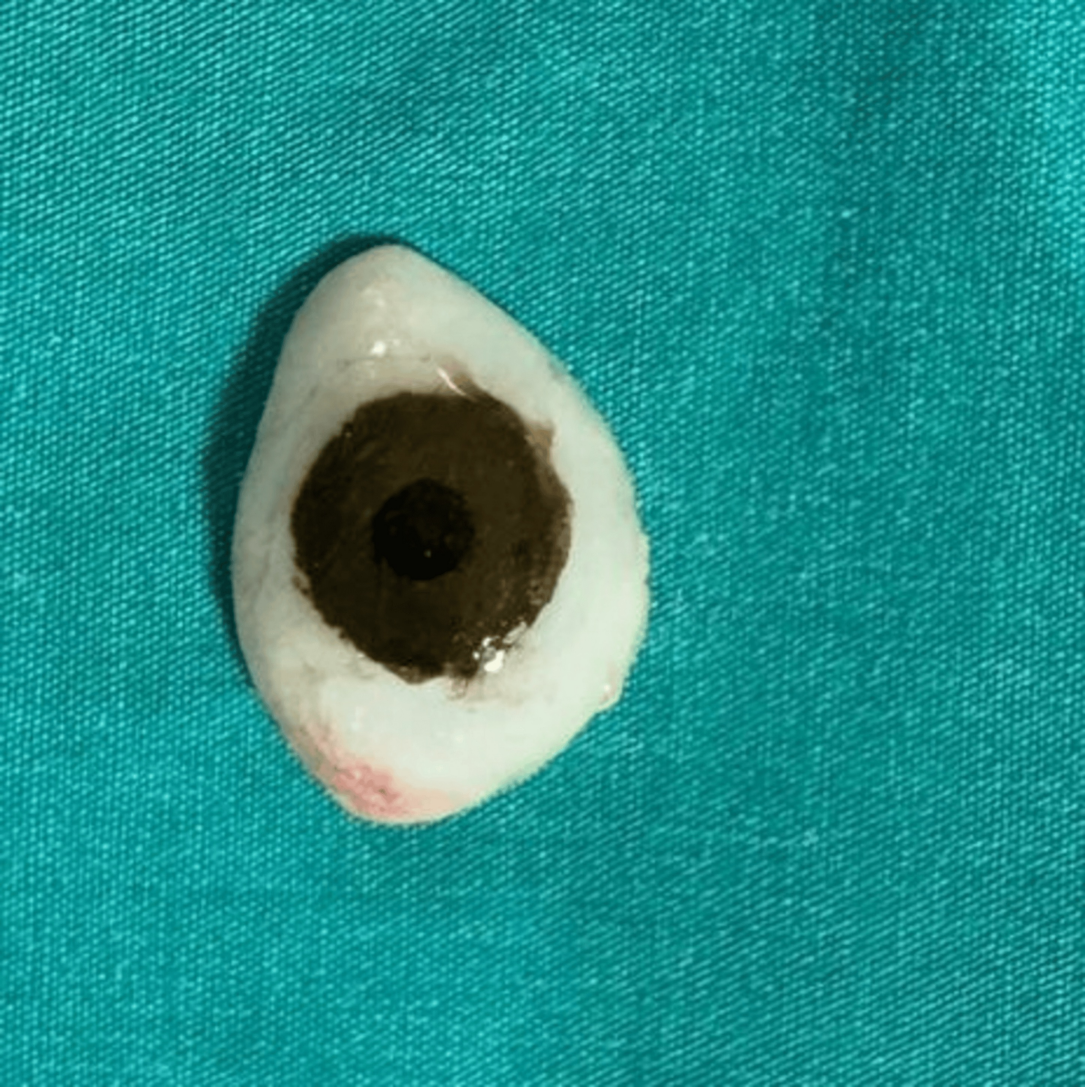
Characterization of the scleral shell prosthesis

The anterior scleral surface of the trial shell was deflasked and reduced by 0.5 mm. A stock curved iris disc was affixed to a close-up photograph of the patient’s iris printed on photo paper. The sclera was then characterized with acrylic paints to simulate the natural brownish hue, and strands of red wool were incorporated to replicate blood vessels, enhancing the realism of the prosthesis. Finally, a layer of transparent heat-cure PMMA was applied, and the scleral shell prosthesis (SSP) was processed in the same curing cycle as described earlier (Figure 6).

Final Finishing and Polishing

**Figure 7:**
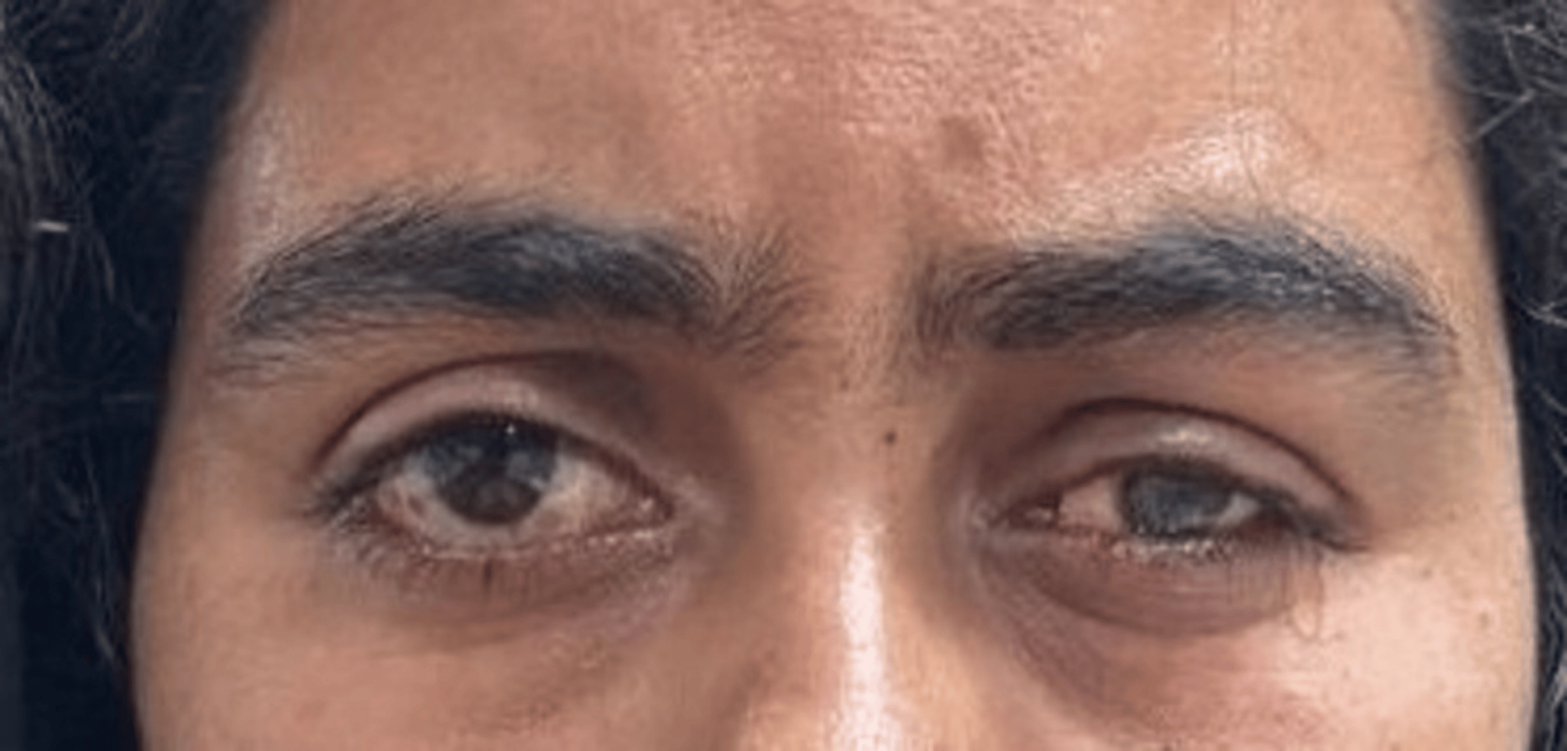
Image after insertion of scleral shell prosthesis Note: Written informed consent to include this image in an open-access article was obtained from the patient.

After curing, the SSP was removed from the flask, and all sharp edges were carefully rounded. The prosthesis was polished using polishing burs, pumice, and a buff to achieve a smooth, glossy surface. The finished prosthesis was then inspected for any irregularities or sharp areas that might cause irritation to the patient (Figure 7).

Maintenance

The patient was instructed on the proper methods of insertion and removal of the prosthesis, with emphasis on regular practice of these techniques. She was advised to remove the prosthesis at night, clean it with antibacterial soap and water, and store it in a container filled with saline solution. Regular recall appointments were scheduled to monitor the prosthesis and perform adjustments as necessary.

## Discussion

Phthisis bulbi, the end stage of ocular disease, presents significant clinical challenges due to its profound effects on orbital anatomy and function. The condition is primarily driven by hypotony and disruption of the blood-ocular barrier, resulting in progressive atrophy, shrinkage, and disorganization of intraocular structures. This leads to a smaller, disfigured globe with thickened sclera and cornea, and in some cases, ossification or calcification. These changes not only compromise aesthetics but also alter orbital volume and symmetry [3]. Managing phthisis bulbi is therefore clinically significant, as it requires addressing both functional impairment and psychological well-being. Custom-made scleral shell prostheses are considered superior to alternatives such as orbital implants or conventional ocular prostheses, particularly in cases involving scleral disfigurement, a reduced palpebral aperture, or strabismus [4].

Their advantages lie in precise customization, uniform pressure distribution, and enhanced aesthetics, features often lacking in stock prostheses or implants. Patient selection depends on factors such as the extent of scleral disfigurement, degree of enophthalmos, and absence of active orbital complications like infection or tumors. Contraindications include severe orbital inflammation, insufficient residual orbital support, and unrealistic patient expectations regarding functional or cosmetic outcomes. Careful evaluation of these factors ensures optimal prosthetic rehabilitation and patient satisfaction [5-7].

Successful management of phthisis bulbi requires attention to prosthetic design, taking into account the unique anatomical and biomechanical challenges of phthisical eyes. Features such as scleral disfigurement, enophthalmos, and potential ossification or calcification influence the prosthesis’ contour, thickness, and overall fit [8].

Biomechanical principles emphasize uniform pressure distribution to minimize orbital deformation and maximize comfort. Material selection is equally critical, with acrylic resin widely used for its biocompatibility, durability, ease of manipulation, and excellent esthetic qualities that closely mimic the natural sclera [9]. Customization remains essential, as each phthisical eye presents distinct anatomical and functional variations. The classification system proposed by Aggarwal et al. provides a structured framework for tailoring prosthetic rehabilitation based on defect characteristics, ensuring that the prosthesis not only restores appearance but also integrates harmoniously with the remaining orbital structures [10]. This individualized approach enhances both patient satisfaction and long-term outcomes in the management of phthisis bulbi.

## Conclusions

This case report highlights the importance of custom scleral shell prostheses in restoring aesthetics and psychological well-being in patients with phthisis bulbi. The step-by-step procedure demonstrates the integration of clinical expertise, biomechanical principles, and advanced materials to address the unique challenges of phthisical eyes. Through precise customization and uniform pressure distribution, these prostheses significantly improve patient quality of life, underscoring the vital role of prosthodontic care in rehabilitation. The report also emphasizes the need for continued interdisciplinary research and innovation to further advance ocular prosthetics and enhance both functional and cosmetic outcomes.
